# The Short-Term Antibacterial Activity of Three Selected Endodontic Sealers against *Enterococcus faecalis* Bacterial Culture

**DOI:** 10.3390/life12020158

**Published:** 2022-01-21

**Authors:** Matej Rosa, Yuliya Morozova, Roman Moštěk, Pavel Holík, Lucia Somolová, Barbora Novotná, Soňa Zábojníková, Kateřina Bogdanová, Kateřina Langová, Iva Voborná, Lenka Pospíšilová, Josef Paul Kovařík

**Affiliations:** 1 Institute of Dentistry and Oral Sciences, Faculty of Medicine and Dentistry, Palacký University, Hněvotínská 3, 775 15 Olomouc, Czech Republic; yulia.morozova@upol.cz (Y.M.); roman.mostek@upol.cz (R.M.); pavel.holik@upol.cz (P.H.); lucia.somolova@upol.cz (L.S.); sona.zabojnikova@upol.cz (S.Z.); iva.voborna@upol.cz (I.V.); lenka.pospisilova@upol.cz (L.P.); josefpaul.kovarik@upol.cz (J.P.K.); 2 Department of Microbiology, University Hospital Olomouc, I. P. Pavlova 6, 779 00 Olomouc, Czech Republic; katerina.bogdanova@fnol.cz; 3 Department of Medical Biophysics, Faculty of Medicine and Dentistry, Palacký University, Hněvotínská 3, 775 15 Olomouc, Czech Republic; katerina.langova@upol.cz

**Keywords:** antibacterial, antimicrobial, endodontic, sealer, poly-epoxy resin, zinc oxide-eugenol, calcium silicate

## Abstract

(1) Background: Microorganisms originating from the microflora of the oral cavity are the main cause of the inflammatory diseases of the dental pulp and periapical periodontium, as well as the failure of endodontic treatment. The subsequent root canal treatment is not able to remove all the pathogens, and a small number of viable bacteria remain in the dentine tubules, which must be sealed by endodontic sealers. These sealers should have at least a bacteriostatic effect to prevent the remaining bacteria from reproducing. The aim of this study is to compare the short-term antibacterial activity of three endodontic sealers based on poly-epoxy resin, zinc oxide-eugenol and calcium silicate with a calcium hydroxide-based sealer. Calcium hydroxide is used as temporary intracanal medicament and, thus, should show significant antibacterial activity. (2) Methods: A total of 25 bovine dentine samples infected with *Enterococcus faecalis* were used in this study. After the sealer placement and a 24 h incubation period, the root canal walls were scraped, and the suspension of dentine fillings was used for a semi-quantitative evaluation of microbial growth. (3) Results: The poly-epoxide resin-based sealer ADSeal™ showed significant antibacterial properties. (4) Conclusions: The highest antibacterial activity was shown in poly-epoxide resin-based sealer group, followed by the zinc oxide-eugenol-based sealer and calcium silicate-based sealer.

## 1. Introduction

Microorganisms and the products of their metabolism play a significant role in the development of inflammatory diseases of the dental pulp and periapical periodontium [1], and they are closely related with the failure of endodontic treatment [2,3] as well. The spectrum of microorganisms is diverse, and it varies in individual cases [4]. A different spectrum of microorganisms is present in cases of dental pulp inflammation and in cases of periapical periodontium inflammation, as well as in cases of primary and secondary root canal infection [5]. What all these inflammatory processes have in common is that they are mainly caused by anaerobic microorganisms [4,5]. The treatment of these inflammatory diseases relies on the mechanical and chemical removal of the microbes from the root canal system [6]. Although the mechanical enlargement of the root canal removes a significant fraction of the microorganisms, it does not ensure the sterility of the root canal system. Therefore, it is recommended to use irrigants with antimicrobial properties as well. The most commonly used irrigant is sodium hypochlorite (NaOCl) [7], which, apart from the antimicrobial action, dissolves vital and necrotic pulp tissue, lubricates the root canal walls and helps to remove the debris from the root canal system [8]. To potentiate the abilities of the NaOCl and to aid the dispersal of the irrigant into the root canal system, where in many cases there are lateral root canals that are inaccessible to mechanical instrumentation and contain residual microbial infection, activation of the irrigant is advised [9]. There are many activation techniques, ranging from manual activation with gutta-percha point or endodontic instruments; mechanical activation, using different types of files mounted on a micro-motor; the negative pressure technique [8], which creates the negative pressure in the root canal by placing the fine suction tip near the working length [10]; laser activation and ultrasonic and subsonic activation [8]. All these procedures, however, are not able to remove all the pathogens, and a small percentage of viable microorganisms still remains in the root canal system [6,11]. The function of the root canal filling is, therefore, to hermetically seal the remaining pathogens in the dentine tubules [12] and, thus, prevent them from communication with the periapical periodontium [13] and reduce their count [14].

The materials used for definitive root canal obturation have to meet multiple requirements, whereas the ideal material should have at least a bacteriostatic effect [7,15,16]. The most ideal material appears to be gutta-percha, which, however, lacks adhesion to the hard dental tissues and antibacterial properties, and, thus, another material called a sealer has to be used. This material fills the gap between the gutta-percha and the wall of root canal [17] and should have at least bacteriostatic effects [16]. Today’s dental market offers a wide variety of endodontic sealers. Based on their composition, chemical reactions and the by-products of their setting and the final products of their setting reactions, there are significant differences in the properties of the different groups of these sealers, including the antibacterial properties. The adequate antibacterial efficacy of endodontic sealers might contribute to the successful and long-lasting outcome of the endodontic treatment and might be the one of many criteria that is taken into consideration when choosing an endodontic sealer.

The aim of this study was to assess the short-term antibacterial effect of three different endodontic sealers on the *Enterococcus faecalis* culture and to compare this with the antibacterial activity of a calcium hydroxide-based sealer. Calcium hydroxide’s antibacterial effect is based on the slow release of hydroxyl anions, which, as highly reactive free radicals, damage the cytoplasmic membrane, cause the denaturation of proteins and damage the DNA of bacterial cells. Its high pH also deactivates enzymes that are important for bacterial metabolism [18]. Because of these properties, calcium hydroxide is often used as temporary intracanal medicament during root canal treatment and was chosen as a negative control group in our study. Although *E. faecalis* is highly resistant to the effect of calcium hydroxide by buffering the alkaline pH up to 11.5 [19], Apexit^®^ Plus (Ivoclar Vivadent, Liechtenstein) calcium hydroxide-based endodontic sealer is capable of increasing the pH levels up to 12.5 [20]. This is above the buffering abilities of *E. faecalis*.

Therefore, the hypothesis of this study was that the calcium hydroxide-based sealers would have the most significant antibacterial activity on the *E. faecalis* culture compared to the other sealers used.

## 2. Materials and Methods

### 2.1. Dentine Specimens

For the purpose of this study, non-carious teeth with simple root and root canal anatomy, preferably single-rooted teeth with one root canal were chosen. In clinical practice, the main reason for tooth extraction is carious destruction. Moreover, the unerupted third molars have more difficult anatomy of the root canal system [21], and extraction of intact teeth because of orthodontic reasons is rare. For these reasons, human teeth were excluded from this study. Bovine teeth were chosen instead, because they are easily available in high numbers, as suggested by Swimberghe et al. [22]. All donors were approximately the same age. According to studies, bovine teeth show similar physical and chemical properties as human teeth [22]. The amount of dentine tubules in bovine teeth is higher, but the diameter of bovine dentine tubules is similar to human dentine, according to Camargo et al. [23]. Although a similar study [24] reported a higher diameter of dentine tubules in bovine dentine than in human dentine specimens, those differences were not statistically significant. Moreover, only the crown dentine of human third molars and the crown and root dentine of bovine incisors were compared in this study.

Before sample preparation, X-ray images were taken to determine the number and patency of the root canals of each tooth. Only single-rooted teeth with one root canal were chosen.

**Figure 1:**
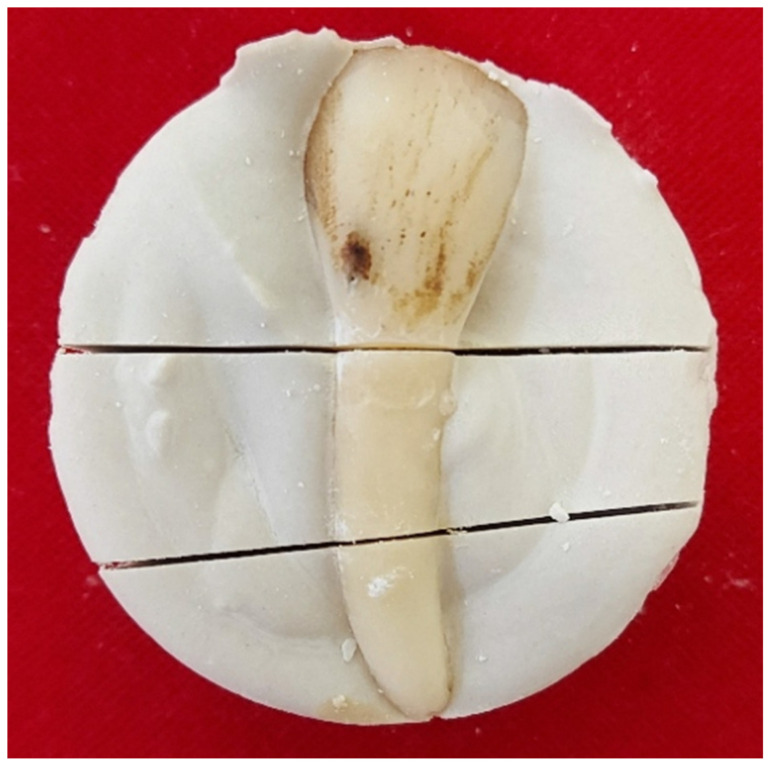
Sectioned bovine incisor fixed in alabaster plaster.

Bovine incisors were fixed in alabaster plaster, the anatomical crown of the tooth was cut away and the root was cut in half, approximately 10 mm long with the dental laboratory saw, as shown in the Figure 1.

**Figure 2:**
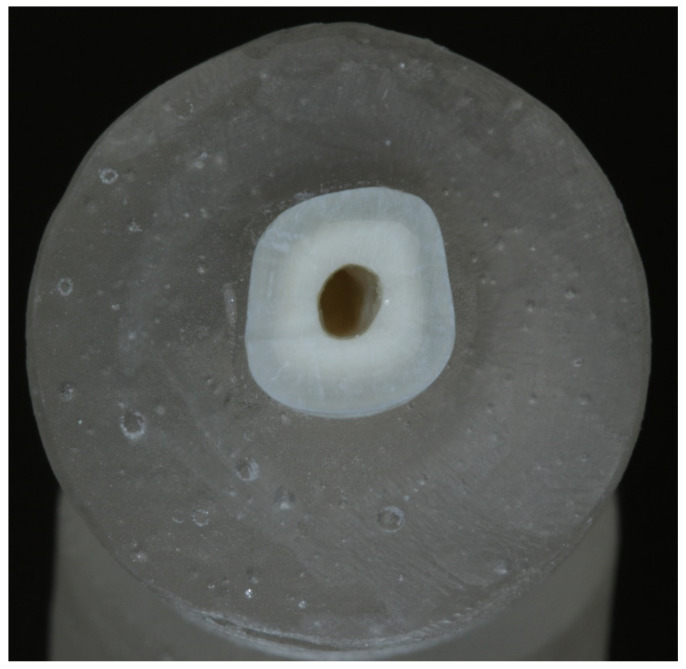
Dentine block ready for bacterial infection.

Both halves were then removed from the plaster and root blocks were created. The root canals of these root blocks were shaped with Gates-Glidden drills No. 1–6 (ISO 50-ISO 150, diameter 0.5–1.5 mm). The common rotary endodontic instruments were not suitable for instrumentation of the large-diameter bovine root canal, since the largest instruments in the sequence have their diameter at the tip equal to 0.5 mm. The root canal was rinsed with 1% sodium hypochlorite between each instrument. After the final instrumentation with Gates-Glidden drill No. 6 was performed, a proper disinfection protocol was followed. This disinfection protocol consisted of rinsing the root canal with 5% sodium hypochlorite for 5 min, followed by a 1 min rinse with 17% EDTA to remove the smear layer and open the dentine tubules. Finally, the disinfection protocol was finished by rinsing the root canal with 1% sodium hypochlorite for 5 min. The root canals were then dried with air and sterile paper points, and the surface of the roots was cleaned with red Sof-Lex™ XT discs (3M ESPE, Monrovia, CA, USA). The surface of the root blocks was coated with Single Bond™ Universal (3M ESPE, Monrovia, CA, USA) and OptiBond™ FL Adhesive (Kerr, Switzerland) to prevent the bacterial penetration of the dentine tubules from the outer walls during incubation, and one end of the root canal was plugged with Filtek™ Ultimate Flowable Restorative composite (3M ESPE, Monrovia, CA, USA). Special care was taken to ensure the root canal walls remained uncoated. This was achieved by filling the root canal with a PTFE band, which was removed after the coating and plugging process was conducted. The plugged end was than sealed in the resin Premacryl™ Plus (SpofaDental, Jičín, Czech Republic), forming a base on which the dentine specimen could stand. The prepared specimens were than sterilized by autoclaving for 20 min at 121 °C and 205 kPa in distilled water. According to this working procedure, 27 specimens were prepared, as shown in Figure 2.

### 2.2. Infection of Root Dentine Blocks

Out of the bacteria which play the most significant part in root canal treatment failure, due to their strong resilience to the currently available endodontic disinfection irrigants and intra-canal medicaments, *Enterococcus faecalis* was chosen.

The standard reference bacterial stain *E. faecalis* CCM 4224 was obtained from the Czech Collection of Microorganisms (CCM) (Faculty of Science, Masaryk University Brno). The bacterial strain was stored in cryotubes (ITEST plus, Králové, Czech Republic) at −80 °C.

*E. faecalis* was inoculated on Columbia blood agar with 5% sheep blood (TRIOS, Czech Republic) and cultivated for 18 ± 2 h at 35 ± 1 °C. The fresh bacterial colonies were used in the bacterial suspension preparation. The colonies were resuspended in 2 mL of saline solution (TRIOS, Hradec Králové, Czech Republic) so the final turbidity was 1, according to McFarland turbidity standard. The final turbidity was measured with a turbidity meter (Erba LaChema, Brno, Czech Republic).

The root blocks were placed into sterile 50 mL centrifuge test tubes (VWR, Stříbrná Skalice, Czech Republic) with 2 mL of sterile distilled water underneath them to prevent desiccation. The sterile 50 mL test tube was filled with 10 mL of brain heart infusion broth (BHI) and a 0.25% solution of glucose to potentiate the growth of biofilm, and 10 µL of bacterial suspension was added. The resulting bacterial concentration was 5 × 10^5^ CFU/mL. The prepared bacterial suspension was pipetted into the root canals of 25 dentine specimens with a micropipette. Five of these specimens were used as positive bacterial growth control specimens. Moreover, two root canals specimens were filled only with BHI without any bacteria. These two specimens were used for sterility control. The specimens were placed into the incubator and cultivated at 35 ± 1 °C for 24 h. The content of the root canal of each specimen was removed with a vacuum pump the next day and the specimens were inoculated with the freshly prepared *E. faecalis* bacterial suspension (5 × 10^5^ CFU/mL) in BHI + 0.25% solution of glucose, with the exception of the sterility control group, which was filled with cultivation broth only. This process was repeated every day, with the exception of weekends, for 14 days. In order to control the positive bacterial growth, the remaining growth medium with bacterial suspension was cultivated alongside the root specimens, followed by positive growth detection (turbidity of growth medium).

### 2.3. Placement of Sealers

In this study, five types of endodontic sealers were used. As a negative control group, the calcium hydroxide-based endodontic sealer Apexit^®^ Plus (Ivoclar Vivadent, Liechtenstein) was chosen. The positive control group consisted of specimens filled only with gutta-percha points and no sealer. The tested sealers were the zinc oxide-eugenol-based sealer Endomethasone N (Septodont, Saint-Maur-des-Fossés, France), the poly-epoxide resin-based sealer ADSeal™ (Meta Biomed, Cheongju, Korea) and the bioceramics-based sealer BioRoot™ RSC (Septodont, Saint-Maur-des-Fossés, France).

The dentine specimens were randomly divided into five groups of five specimens. In similar accessible studies, the number of specimens in each group ranged from five up to ten [14,25,26]. According to the results of the statistical analysis, the number of specimens was sufficient. In each group, the content of the root canals was removed, and the root canals were rinsed out with a sterile saline solution and dried with sterile paper points. The sealers were prepared according to the manufacturers’ instructions. The placement of the sealers followed the manufacturers’ instructions as well, since this would create similar conditions in the filled root canal as in the real situation. The sealers were placed in the root canal with help of the sterile gutta-percha point or the mixing cannula provided by manufacturer. Sterile gutta-percha point was then used to evenly disperse the sealer and was left in the root canal. The excess of the gutta-percha point was cut away and the top of the root canal was sealed with Single Bond™ Universal (3M ESPE, Monrovia, CA, USA) and Filtek™ Ultimate Flowable Restorative composite (3M ESPE, Monrovia, CA, USA). The specimens were incubated at 35 ± 1 °C for 24 h to observe the short-term antibacterial activity. This time period was chosen because of the relatively long setting times of the endodontic sealers that were used [15,27,28].

### 2.4. Testing the Antibacterial Activity

After the incubation period was over, the specimens were rinsed in sterile saline. The composite seal was removed using dental diamond burrs, and the gutta-percha and sealer were removed by an H-file ISO 80 (diameter 0.8 mm at the tip, taper 2%). The walls of each root canal were scraped with a sterile rotary instrument (Peeso Largo Reamer number 5, diameter 1.5 mm), and the filings containing bacteria were placed in 2 mL micro-tubes (Eppendorf, Saxony, Germany) containing 1 mL of sterile saline solution. This suspension was thoroughly mixed and 10 µL of suspension were immediately inoculated on Mueller-Hinton cultivation agar (TRIOS, Hradec Králové, Czech Republic) and diluted with a sterile inoculation loop for the semi-quantitative evaluation of microbial growth. This ensured, that a minimal amount of filings, which could have contained sealer particles as well, were inoculated on agar plates. This procedure was used in similar accessible studies [14,26]. The agar plates were cultivated at 35 ± 1 °C for 18 ± 2 h. After the incubation period was over, the log CFU/mL of bacteria was evaluated. The measured values are shown in Table 1. The detection limit of bacterial growth was 10^2^ CFU/mL.

### 2.5. Statistical Analysis

The quantitative variables are presented as means, standard deviations (SD) and minimum and maximum values. To compare more independent specimens, an analysis of variance (ANOVA) was used. In the case of statistically significant results, Dunnett’s post hoc test was performed to compare each sealer with the positive and negative control groups.

All tests were performed at the level of statistical significance α = 0.05. Results where the *p*-value was less than 0.05 were considered statistically significant and are marked red in tables.

The data were analyzed with IBM SPSS Statistics for Windows, Version 23.0. (IBM Corp., Armonk, NY, USA), MedCalc Statistical Software version 19.1.5 (Ostend, Belgium) and TIBCO STATISTICA version 13.4.0.14 software.

## 3. Results

The ANOVA comparison of the mean values of specimens with the negative control group is shown in Table 2.

The ANOVA did not prove that there were any statistically significant differences between the mean log CFU values depending on the type of sealer, *p* = 0.179.

The ANOVA comparison of the mean values of the specimens with the positive control group is shown in Table 3.

The ANOVA proved that there were statistically significant differences between the mean log CFU values depending on the type of sealer, *p* = 0.049.

Dunnett’s post hoc tests were performed to compare each specimen’s value with the positive control group. The results are shown in Table 4.

According to Dunett’s post hoc tests, a statistically significant difference between positive control group (mean value = 4,400,020) and ADSeal group (mean value = 40,600) was proven, *p* = 0.043.

**Figure 3:**
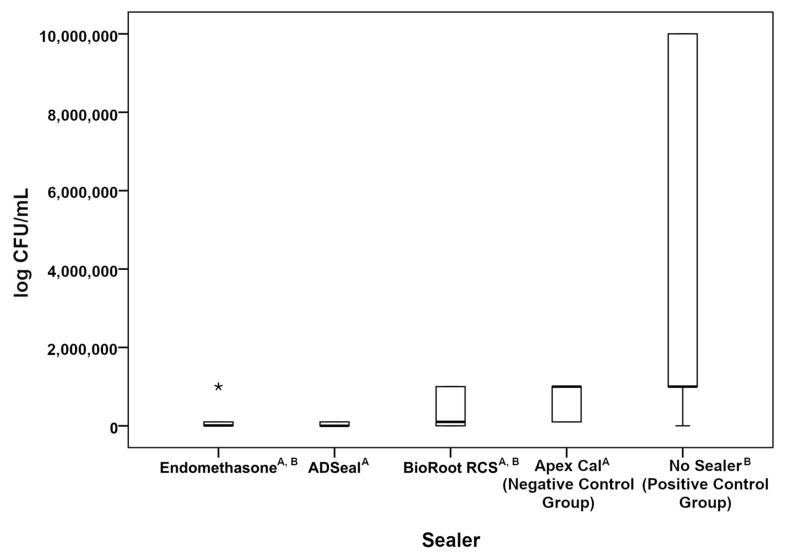
Distribution of quantitative values of each sealer group. Groups sharing the same letters do not represent statistical differences. The “*” sign in the graph represents the extreme outlier value.

The distribution of quantitative values is shown by box graph (Figure 3).

The horizontal line in graph represents median value, the bottom edge of the box represents the value of the 1st quartile (25th percentile) and the upper edge represents the value of the 3rd quartile (75th percentile). The brackets represent the maximum and minimum measured values.

## 4. Discussion

The aim of this study was to determine the antibacterial activity of the three selected endodontic sealers against *E. faecalis* bacterial culture and compare this with the antibacterial activity of calcium hydroxide, represented in this study by the endodontic sealer Apex Cal (Ivoclar Vivadent, Liechtenstein). *E. faecalis* was chosen for this study because of its high resistance against endodontic rinsing and disinfecting agents. It is also the most common bacterial strain isolated from teeth in which the primary endodontic treatment has failed [5]. A statistical analysis of the results has shown no statistically significant differences between the antibacterial activity of the tested sealers and the negative control group. Thus, the data analysis shows that all studied endodontic sealers were able to significantly reduce the number of viable bacterial cells, similar to the negative control group.

By comparing the results of the antibacterial activity of the tested endodontic sealers with the mean log CFU/mL values of the positive control group, statistically significant differences between mean log CFU/mL, according to the type of endodontic sealer, were found, whereas a statistically significant difference between the positive control group and ADSeal (Meta Biomed, Cheongju, Korea) sealer was proven. By evaluating the results shown in Figure 2, it is possible to rank the individual endodontic sealers according to their increasing antibacterial activity as follows: BioRoot RCS (Septodont, Saint-Maur-des-Fossés, France), Endomethasone N (Septodont, Saint-Maur-des-Fossés, France) and ADSeal (Meta Biomed, Cheongju, Korea).

BioRoot RCS (Septodont, Saint-Maur-des-Fossés, France) is a two-component calcium silicate-based endodontic sealer consisting of powder and liquid. The powder contains tricalcium silicate and zirconium oxide, which serve as a radiopaque filler, and povidone, which enhances adhesion to hard dental tissues. The liquid contains mostly sterile water and calcium chloride, which serves as setting modifier [27]. The antibacterial activity of this endodontic sealer is based on the chemical reaction of its setting, where the reaction of tricalcium silicate with water forms a calcium silicate hydrate gel and calcium hydroxide, according to the chemical equation [29]:

3CaO·SiO_2_ + H_2_O → C–S–H + Ca(OH)_2_ (1)

The environment formed during this chemical reaction has high pH values, which is the source of the antibacterial properties of this sealer. This explains its similar antibacterial effect on *E. faecalis* culture as the negative control group represented by the calcium hydroxide-based sealer Apex Cal (Ivoclar Vivadent, Liechtenstein). The lower antibacterial efficacy of the calcium silicate-based sealer compared to other tested sealers may be due to the high resistance of *E. faecalis* bacterial culture to calcium hydroxide, especially when organized in a biofilm [30]. An alkaline environment enhances the adherence of *E. faecalis* to the collagen fibers of root canal walls, which increases its infectivity and the risk of residual infection [31]. *E. faecalis* is able to buffer the highly alkaline pH by activating its proton pump. However, it is only able to buffer the alkaline pH until its value reaches 11.5 [19]. The pH value of BioRoot RCS (Septodont, Saint-Maur-des-Fossés, France) sealer lays between 11.25 [32], which is slightly below the limit of the buffering abilities of *E. faecalis*, and 11.7 [27]. This difference in pH may be due to the different mixing ratio of powder and liquid during the sealer preparation, which may ultimately affect its antibacterial properties.

The resulting short-term antibacterial activity of calcium silicate-based sealer proved in this study is in high contrast with the results from different studies [25,32,33,34]. In studies where BioRoot RCS (Septodont, Saint-Maur-des-Fossés, France) sealer was used [25,32], the short-term antibacterial activity of calcium silicate-based sealer against the *E. faecalis* culture was significantly higher compared to the poly-epoxide resin based sealer. This difference may be caused by the different methodology of the study. Alsubait et al. [25] used horizontally sectioned roots of human teeth in their study and the BioRoot RCS (Septodont, Saint-Maur-des-Fossés, France) sealer was placed on these samples with a syringe. This resulted in a relatively even distribution of the sealer on the entire wall of the root canal, which is not possible to achieve under the real conditions of a root canal treatment [35]. The placement of the BioRoot RCS (Septodont, Saint-Maur-des-Fossés, France) sealer according to manufacturer’s instructions with sterile gutta-percha point may have led to the formation of spots on the root canal wall, which were not coated with sealer. In these non-coated places of root canal wall, no antibacterial activity was shown.

The study of Colombo et al. [32] tested the antibacterial activity of BioRoot RCS (Septodont, Saint-Maur-des-Fossés, France) using the agar diffusion test (ADT) and the direct contact test (DCT). However, the absence of dentine in this study may have led to different results. Dentine is able buffer the alkaline environment created during the setting reaction of calcium silicate-based sealers and, thus, influence their antibacterial effect [36].

Other studies [33,34] have used EndoSequence BC (Brasseler, Savannah, GA, USA) sealer as the representative for the calcium silicate-based sealer. Unlike BioRoot RSC (Septodont, Saint-Maur-des-Fossés, France) sealer, it is apremixed sealer in a syringe, which may contribute to its stable pH, which is, in this case, nondependent on the mixing ratio of powder and liquid. The different composition of these two sealers may contribute to the higher antibacterial activity of EndoSequence BC (Brasseler, Savannah, GA, USA) sealer as well. The aluminum oxide and silicon dioxide present in EndoSeqence BC (Brasseler, Savannah, GA, USA) sealer damage the cell wall of Gram-positive bacteria, thus allowing better permeability of calcium hydroxide into the cytosol of the bacterial cell, where it could denature the bacterial DNA and, thus, contribute to the higher antibacterial effect of this sealer [34]. Further studies are needed to identify the possible by-products of the setting reaction of both sealers.

Endomethasone N (Septodont, Saint-Maur-des-Fossés, France) is a two-component sealer consisting of a powder containing hydrocortisone acetate, thymol iodide, barium sulphate, zinc oxide and magnesium stearate and a liquid containing eugenol [37]. The antibacterial action of this endodontic sealer is based on the antibacterial effect of its components. The free hydroxyl groups of eugenol can alter the composition of the bacterial cytoplasm membrane, prevent the transport of ATP and ions and inhibit some of the bacterial enzymes [38]. Eugenol is released from this sealer during its setting phase, but due to the inaccurate mixing ratio, free eugenol might be released even after the setting of the sealer [39]. Thymol, which is present in this sealer, may affect the *E. faecalis* culture as well [40]. Even though this type of sealer has numerous evidenced disadvantages, such as poor sealing ability [41], high cytotoxicity [42] and discoloration potential [43], it is still used in clinical practice, due to its low price and easy manipulation. Therefore, this type of sealer was investigated as well.

The results of the short-term antibacterial activity obtained in this study correspond with the results of similar studies performed in the past [26,44]. The study of Prestagaard et al. [44] proved that the zinc oxide-eugenol-based sealer is able to significantly decrease the number of viable bacterial cells of *E. faecalis* culture compared with a calcium hydroxide-based sealer. However, this significant antibacterial effect of zinc oxide-eugenol-based sealers decreases over time.

Other studies [45,46] have used the ADT and DCT to determine the antibacterial activity of zinc oxide-eugenol-based sealers. Both studies have shown higher antibacterial activity of zinc oxide-eugenol-based sealer than calcium hydroxide-based material. This further confirms the claim that the antibacterial activity of these sealers is based on their main components and not on the products and by-products of their setting reaction, nor their setting pH value, which lays slightly below the neutral pH values [47].

ADSeal (Meta Biomed, Cheongju, Korea), the poly-epoxide resin-based sealer, had the most significant effect on the *E. faecalis* bacterial culture in this study. This corresponds with results of similar studies performed on dentine blocks [26,44]. In both of these studies the high antibacterial effect of poly-epoxide resin-based sealer on *E. faecalis* culture was proven compared to a calcium hydroxide-based sealer. The study of Prestagaard et al. proves the significant short-time antibacterial effect of poly-epoxide resin-based endodontic sealers, which decreases over time [44].

Studies testing the antibacterial activity of poly-epoxide resin-based endodontic sealers using the ADT and DCT [45,46] have shown significant antibacterial effects of this group of sealers against *E. faecalis* culture in comparison with the effect of a calcium hydroxide-based root canal sealer.

The antibacterial effect of ADSeal (Meta Biomed, Cheongju, Korea) sealer is probably based on the antibacterial action of its main components and the by-products of its setting. It is a two-component sealer, consisting of a base and a catalyst. The base of this sealer consists, mainly, of bisphenol A diglycidyl ether [48], which has mutagenic effects [49]. During the setting reaction of this sealer, a minimal amount of formaldehyde is produced, which reacts with bacterial proteins, DNA and RNA and, thus, negatively affects the *E. faecalis* culture [34,50]. This statement is supported by the results of the DCT and ADT studies [45,51] in which the already set poly-epoxide resin-based endodontic sealer was not able to significantly affect the *E. faecalis* culture. The pH values during the setting reaction of this sealer have no effect on its antibacterial effect, since the pH values lay slightly below the neutral pH values [52]. The formation of a minimal amount of formaldehyde during the setting reaction of this sealer can be deducted from the similar composition of this sealer and another sealer of poly-epoxide resin-based sealer group, AH Plus (Dentsply DeTrey, Germany) [48], in which the minimal amount of formaldehyde formation during its setting reaction was proven [53].

The limits of this study lay, foremost, in the low number of studied specimens, the different diameters of the root canals and, thus, different widths of dentine. In spite of an X-ray examination, there could be some variations regarding the anatomy and histology of the selected teeth. This fact could not be influenced by the researchers, however. This may have resulted in an uneven distribution, as well as an uneven amount of sealer in each root canal, which may have led to non-constant results throughout the entire group of specimens. The different methodologies of sealer placement of the different groups may have contributed to the uneven distribution of the individual sealers on the root canal walls. However, this was the working procedure recommended by the manufacturer of each of the sealers used in this study.

The cited literature shows that some sealers are able to maintain their antibacterial ability over time. The study of Alsubait et al. [25] proved that the antibacterial activity of the calcium silicate-based sealer BioRoot RCS (Septodont, Saint-Maur-des-Fossés, France) increased 28 days after its setting. Further studies based on the comparison of the short-term and the long-term antibacterial activity of sealers used in this study against *E. faecalis* culture are planned in the future.

## 5. Conclusions

The highest short-term antibacterial activity of the tested sealers was shown in the poly-epoxide resin-based sealer ADSeal (Meta Biomed, Cheongju, Korea), due to its content of bisphenol A diglycidyl ether, as well as a minimal amount of formaldehyde production during its setting reaction. The second highest short term antibacterial activity was shown in the zinc oxide-eugenol-based sealer Endomethasone N (Septodont, Saint-Maur-des-Fossés, France), due to the release of free eugenol during the setting phase, as well as the antibacterial effect of the thymol iodide contained in the powder of this sealer. The calcium silicate-based endodontic sealer BioRoot RCS (Septodont, Saint-Maur-des-Fossés, France) showed the lowest antibacterial properties comparable to the calcium hydroxide-based endodontic sealer Apexit^®^ Plus (Ivoclar Vivadent, Liechtenstein). This may have been caused by the different mixing ratio of powder and liquid, which could have affected the pH of this sealer and, thus, its antibacterial properties.

## Figures and Tables

**Table 1:** Measured values of log CFU/mL for each dentine specimen.

Sealer	Specimen Number				
	1	2	3	4	5
Endomethasone	1 × 10^6^	1 × 10^5^	1 × 10^3^	1 × 10^2^	1 × 10^4^
ADSeal	1 × 10^3^	1 × 10^3^	1 × 10^5^	1 × 10^3^	1 × 10^5^
BioRoot RCS	1 × 10^6^	1 × 10^2^	1 × 10^2^	1 × 10^5^	1 × 10^6^
Apex Cal (Negative Control Group)	1 × 10^6^	1 × 10^6^	1 × 10^5^	1 × 10^6^	1 × 10^5^
No sealer (Positive Control Group)	1 × 10^6^	1 × 10^6^	1 × 10^7^	1 × 10^2^	1 × 10^7^

**Table 2:** ANOVA comparison of the mean values of specimens with the negative control group.

Sealer	N	Mean	SD	Minimum	Maximum	*p*
Endomethasone	5	222,220	436,804.4	100	1,000,000	0.179
ADSeal	5	40,600	54,224.5	1000	100,000	
BioRoot RCS	5	420,040	530,997.2	100	1,000,000	
Apex Cal (Negative Control Group)	5	640,000	492,950.3	100,000	1,000,000	

**Table 3:** ANOVA comparison of the mean values of the specimens with the positive control group.

Sealer	N	Mean	SD	Minimum	Maximum	*p*
Endomethasone	5	222,220	436,804.4	100	1,000,000	0.049
ADSeal	5	40,600	54,224.5	1000	100,000	
BioRoot RCS	5	420,040	530,997.2	100	1,000,000	
No sealer (Positive Control Group)	5	4,400,020	5,128,331.1	100	10,000,000	

**Table 4:** Comparison of the values of each specimen with the positive control group, according to Dunnett’s post hoc tests.

No Sealer (Positive Control Group) vs	*p*
Endomethasone	0.054
ADSeal	0.043
BioRoot RCS	0.068

## Data Availability

Not applicable.
